# Structure Elucidation and Immunostimulatory Activity Evaluation of a Galactoglucan from *Alpinia officinarum* Hance

**DOI:** 10.3390/foods13244019

**Published:** 2024-12-12

**Authors:** Zhou Xu, Yanxia Xiong, Pei Hu, Long Chen, Jianhua Wan, Chenggang Huang, Wenjun Liu

**Affiliations:** 1 Shanghai Institute of Materia Medica, Chinese Academy of Sciences, 501 Hai Ke Road, Shanghai 201203, China; xz_619@163.com; 2 State Key Laboratory for the Modernization of Classical and Famous Prescriptions of Chinese Medicine, No.1899 Meiling Road, Nanchang 330103, China; xiongyx@crjz.com (Y.X.); hupei@crjz.com (P.H.); chenlong380@crjz.com (L.C.); wanjianhua@crjz.com (J.W.); 3 School of Chemical Engineering, East China University of Science and Technology, 130 Mei Long Road, Shanghai 200237, China

**Keywords:** *Alpinia officinarum* Hance, galactoglucan, immunostimulatory activity

## Abstract

*Alpinia officinarum* Hance has a medicinal history of thousands of years in treating cough, diabetes, and gastrointestinal system diseases, and it is also a medicine food homology (MFH) plant in China. To evaluate the pharmacological activities of polysaccharides from the rhizomes of *A. officinarum*, polysaccharides were initially obtained by hot-water extraction and the ethanol precipitation method. A homogenous polysaccharide designated as AOP-w was isolated by a DE-52 column. The proposed structure was elucidated and the immunoregulatory effects on RAW 264.7 macrophage cells were evaluated. The results showed that AOP-w had a molecular weight of 5.26 kDa, and mainly consisted of galactose and glucose (molar ratio of 0.12:0.88). Its backbone comprised α-(1→4)-Glc*p*, α-(1→4,6)-Glc*p* and β-(1→3,4)-Gal*p* residues, terminated by α-(1→6)-Glc*p* and T-Glc*p* residues. AOP-w was nontoxic to RAW 264.7 cells, but demonstrated promotion in cell proliferation within a 100 μg/mL concentration. The immunostimulatory effects of AOP-w were confirmed by the elevated NO production of AOP-w-treated cells. Moreover, the RNAseq was conducted and the results showed that AOP-w may activate the TNF and NF-κB signaling pathways by binding to Toll-like receptors, thereby affecting the immune modulatory activity of RAW264.7 cells. These results suggest a high potential of AOP-w from *A. officinarum* for immunotherapeutic applications.

## 1. Introduction

Plants exhibit vast potential in drug discovery and nutraceuticals, especially medicinal plants in folk medicine. In China, herbal medicines have a long history use and pronounced therapeutic efficiency in various diseases, especially chronic disease. *Alpinia officinarum* Hance (*A. officinarum*) belongs to the foremost species *Alpinia* of the *Zingiberaceae* family, which is a highly endowed medicinal plant [1]. It is a herbaceous perennial plant commonly known as “lesser galangal” that originates from China, and is widely planted in Guangdong, Guangxi, Taiwan and other regions [2]. The rhizomes of *A. officinarum* were traditionally used in China for many years to treat cold and relieve pain, cease vomiting, and warm and strengthen the stomach [3]. Modern studies have suggested that *A. officinarum* possesses various biological activities [4]*,* including anti-inflammatory [5,6], anti-obesity [7], anticancer [8,9], immunoregulation [10] and diabetes amelioration [11,12]. Moreover, A. officinarum is currently in clinical use when prescribed within the TCM formula. For instance, the classical Piji pill is clinically used for accumulation and stagnation treatment, and the Hua Zheng Hui Sheng tablet is for stomach cancer management. Similarly, the Liangfu pill is clinically used for peptic ulcers and stomach aches, and the Anzhong tablet contains *A. officinarum* for the treatment of gastritis and gastroesophageal disease [13]. Therefore, *A. officinarum* has great potential for drug development.

Recent studies have shown that the flavonoid compounds isolated from *A. officinarum* are responsible for its anticancer and pain alleviation activities [14]. Diarylheptanoids are the most commonly isolated molecules from *A. officinarum* and are considered the major active components of *A. officinarum* [15]. Besides medicinal application, *A. officinarum* has also been explored as a spice in culinary preparations, and recognized as a common medicine and food homologous in many countries. The analysis of the nutrient composition of *A. officinarum* showed that the rhizome contained galangin, kaempferide, quercetin and volatile oil. The spicy component of *A. officinarum* is galangol, which has the functions of warming the spleen and stomach, and relieving pain. Regardless of the small molecules within the rhizomes of *A. officinarum*, the biomacromolecules, especially the polysaccharides, should also be noticed. *A. officinarum* is usually prepared as a decoction, [1] and polysaccharide is the most abundant constituent in decoction. Therefore, further investigation on the polysaccharide from this plant possesses great medicinal value and clinical significance.

Polysaccharides from herbal medicines have demonstrated a wide spectrum of biological properties [16], including gut microbiota regulation [17], anti-tumor [18], immunostimulation [19], anti-inflammation [20] and anti-oxidation [21]. Recently, several polysaccharides from the *Zingiberaceae* family have been discovered to possess immunoregulatory activity [22]. Hence, it is essential to assess the immunostimulating activities of the polysaccharides from *A. officinarum*. However, rare studies are conducted on polysaccharides from *A. officinarum*. Although Ni et al. (2022) [10] and Wen et al. (2024) [23] reported a water-soluble polysaccharide and an acidic polysaccharide from *A. officinarum*; however, accumulating data are still needed for the fine-structure elucidation and various mechanism revelation.

Thus, in this study, we extracted polysaccharide from the rhizomes of *A. officinarum* by a traditional hot-water extraction and the ethanol precipitation method. The structural information was acquired, including homogeneity, monosaccharide constitution, the functional groups, the glycosidic linkages type and the fine structure. Furthermore, the immunoregulatory activities of the polysaccharide on RAW 264.7 cells were evaluated and the RNAseq was exploited to reveal the mechanism. These results will provide new insights for studies on the pharmacological mechanisms of polysaccharides within *A. officinarum*.

## 2. Materials and Methods

### 2.1. Extraction and Purification

Initially, the dried rhizomes of *A. officinarum* (Zhanjiang, Guangdong, China) were defatted with 90% alcohol twice, and then extracted with 10 volumes (*v*/*w*) of hot water for 2 h and 8 volumes (*v*/*w*) of water for 1.5 h, respectively. The combined aqueous fractions were filtered and concentrated to 1 g/mL of crude drug. Subsequently, the concentrated solution was precipitated by adding absolute ethanol until the ethanol concentration reached 80%. After centrifugation, the precipitate was dried at 60 °C and used for subsequent purification.

The polysaccharide was then redissolved in ddH_2_O and subjected onto a preparative DE-52 column (Borui Saccharide, Yangzhou, China). The samples were eluted with three-fold volume eluents of 0–1.0 M NaCl at 15 mL/min. Elutes were detected by the phenol-sulfuric acid method after scanning at 490 nm by a microplate reader. The scatter of the tube number versus absorption was drawn and the polysaccharides were collected according to the peak position. After freeze-drying, each fraction was collected and renamed as AOP-w, AOP-0.2, AOP-0.5 and AOP-1.0.

### 2.2. Molecular Weight Analysis

AOP-w and pullulan standards were dissolved in the mobile phase at 5 mg/mL, respectively. After centrifugation, the supernatant was collected and the molecular weight was determined by using high-performance gel permeation chromatography (HPGPC) equipped with a RI-20A detector (Shimadzu, Japan) and a tandem BRT105-103-101 column (8 × 300 mm, Borui Saccharide, Yangzhou, China). The prepared AOP-w and standard solutions were subjected to the HPGPC system. The mobile phase was 0.05 M NaCl and eluted at 0.7 mL/min. The chromatograms were recorded and the data were analyzed.

### 2.3. FT-IR Analysis

AOP-w (1 mg) was accurately weighted and 198 mg of potassium bromide (Tianjin Tianguang, Tianjin, China) was added, mixed, compressed into tablets and then analyzed using FT-IR650 (Gangdong, Tianjin, China). The data were scanned and recorded within the range of 400–4000 cm^−1^.

### 2.4. Monosaccharide Composition Analysis

The AOP-w sample and the monosaccharide standards were dissolved in a 3 M trifluoroacetic acid (TFA) solution at a proper concentration. Then, the solution was put into a metal bath at 120 °C for saccharide hydrolysis. After a 3 h reaction, the solution was dried and redissolved in ddH_2_O. After centrifugation, the supernatant was analyzed by the high-performance anion exchange chromatography (HPAEC, ICS5000, ThermoFisher, Waltham, MA, USA) system equipped with an electrochemical detector. The Dinoex Carbopac PA20 (3 × 150 mm) column was adopted. The detailed performance of the HPAEC was referred to in the previous method [24].

### 2.5. Methylation Analysis

AOP-w was methylated, hydrolyzed and acetylated to partially obtain the *O*-methylated alditol acetates (PMAAs) derivatives according to the literature [25]. The derivatives were analyzed by gas chromatography–mass spectroscopy (GC-MS, 1300-7000 ThermoFisher, Waltham, MA, USA). The column was HP-INNOWAX (30 m × 0.32 mm × 0.25 μm, Agilent, Palo Alto, CA, USA). The temperature programming condition was set as the initial temperature was 140 °C, with increment of 1 °C/min to 230 °C. The sample inlet was set at 250 °C and the temperature for the detector was 250 °C. The gas was He at a flow rate of 1 mL/min. They were identified by their typical retention time according to the standards in the lab and electron impact spectra.

### 2.6. Nuclear Magnetic Resonance (NMR) Spectroscopy

AOP-w was firstly dissolved in deuterium oxide (D_2_O, Aladdin, Shanghai, China), and then under freeze-drying. This procedure was repeated three times. Then, the dried AOP-w was prepared in D_2_O at 80 mg/mL and subjected to the 600 MHz NMR spectrometer (Avance, Bruker, Rheinstetten, Germany). One-dimensional and two-dimensional NMR spectra were obtained. The data were processed with Bruker TopSpin 4.1.3 software. Chemical shifts were referenced to the internal standard of d6-acetone (δ_C_ 30.89).

### 2.7. Cell Culture

RAW 267.4 macrophage cells were cultured in a high-glucose DMEM medium, supplemented with 10% fetal bovine serum and 1% penicillin/streptomycin. The cells were maintained in a humidified atmosphere of 5% CO_2_ at 37 °C. Exponential-phase cells were used for the bioactivity study.

### 2.8. Cell Viability

RAW 264.7 cells were cultured in a 96-well plate at a concentration of 5 × 10^4^ cells per well. After attachment to the plate, cells were treated with AOP-w in the range from 0 to 800 μg/mL. After 24 h treatment, MTT at 5 mg/mL was added for an additional 4 h incubation. Then, the old medium was discarded and 100 μL of DMSO was added to each well. After incubation in the dark for 15 min, the mixture was scanned at 570 nm by a microplate reader (Epoch, Biotek, Winooski, VT, USA). Cell viability was calculated by the comparison of the absorption value for the treated groups to the medium control group.

### 2.9. Neutral Red Phagocytosis Analysis

After overnight attachment, RAW 264.7 cells were treated with AOP-w (0–800 μg/mL) for 24 h. Subsequently, cells were rinsed with PBS twice. Then, 0.1% neutral red solution was added and incubated for an additional 1 h. After that, the supernatant was removed. The absorption of the cells was determined after cell lysis and the phagocytosis index was calculated.

### 2.10. Nitric Oxide (NO) Assay

RAW 264.7 cells at the exponential phase were plated into a 96-well plate at a density of 5 × 10^3^ well/200 μL. After treatment with AOP-w for 24 h, the supernatant was collected and 100 μL Griess reagent was added to each sample. After incubation for 10 min, the absorption of the mixture was assayed by a microplate reader (Epoch, Biotek, Winooski, VT, USA) at 492 nm. The standard NaNO_2_ was adopted to draw the standard curve for the produced NO content assay.

### 2.11. RNA Sequencing Analysis

RAW 264.7 cells were inoculated at a density of 5 × 10^5^ cells/100 μL. After attachment, cells were treated with the medium as the control and 200 μg/mL AOP-w for 24 h. Then, cells were collected for transcriptome analysis. Total RNA was extracted with TRIzol reagent, and the RNA concentration and integrity were assessed with a NanoDrop2000 spectrophotometer. Sequencing libraries were prepared with the TruSeqRNA kit. Sequencing libraries were then prepared by IlluminaHiSeqxten/NovaSeq6000 (2 × 150 bp read length). Differentially expressed genes (DEGs) were identified using DESeq2 package in R 4.3.1 software, selecting genes with a *p*-value < 0.05 and a fold-change FC > 1.5. Gene Ontology (GO) enrichment analysis of DEGs was conducted using the topGO package (https://www.geneontology.org/ (accessed on 6 September 2024)). Additionally, the KEGG database was employed to analyze the DEGs involved in significant biochemical metabolic pathways and signal transduction pathways (https://www.kegg.jp/ (accessed on 6 September 2024)).

### 2.12. Statistical Analysis

All results were conducted in triplicate, and the data were displayed as the mean ± standard deviation (SD). The statistical significance of the differences was analyzed by GraphPad Prism (v9.0), and a one-way analysis of variance (ANOVA) was employed to determine the statistical difference among the groups. *p*-value < 0.05 was considered to be statistically significant.

## 3. Results and Discussion

### 3.1. Homogenous Polysaccharide Obtained from A. officinarum

**Figure 1:**
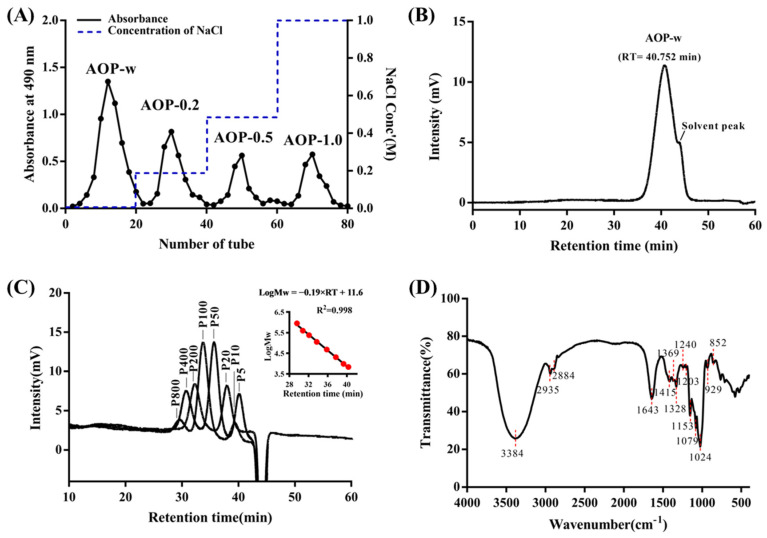
Purification and chemical characterization of polysaccharides from *A. officinarum* (AOP). (**A**) Elution curves of polysaccharides from the DE-52 column after eluting with different concentrations of NaCl and determined by colorimetric phenol–sulfuric acid method. (**B**) HPGPC chromatogram of AOP-w with an RI detector. (**C**) HPGPC chromatograms of pullulan standards for molecular weight determination and the linear regression plot in the upper panel with each standard Mw in red dot. (**D**) FT-IR spectrum of AOP-w.

As shown in Figure 1A, the AOPs fractionation on a DE-52 column yielded four distinct peaks, which were designated as AOP-w, AOP-0.2, AOP-0.5 and AOP-1.0. After collection, dialysis and freeze-drying, the yields of the four fractionated AOPs were 13.8%, 6.4%, 2.0% and 2.5%, respectively. Then, the AOP-w with highest yield was further purified and characterized. The peak of AOP-w in the HPGPC chromatogram was relatively symmetric and sharp at 40.75 min (Figure 1B), indicating the homogeneity of the AOP-w. The regression analysis was conducted on the weight–average molecular weight (Mw) of the pullulan standards versus their retention time. The result of regression analysis is presented in Figure 1C. The high R^2^ value (0.998) indicates that the proposed linear regression model could successfully predict the molecular weight of the AOP-w. Thus, the weight–average Mw of AOP-w after calculation was 5.26 kD. The number–average molecular weight (Mn) was predicted to be 5.25 kDa and the polydispersity index (PDI, Mw/Mn) value was 1.0, indicating a good distribution of the AOP-w we obtained. Therefore, AOP-w in this study was a polysaccharide with relatively low polymerization and high homogeneity.

### 3.2. Functional Groups of AOP-w

As shown in Figure 1D, the infrared spectra of AOP-w exhibited identical absorption peaks. A wide and intense absorption band from 3600 to 3200 cm^−1^ in AOP-w was observed to be apportioned to the stretching vibration of O-H [26]. Moreover, a high absorption band at 1643 cm^−1^ was due to the absorbed water. The peaks at 1024 cm^−1^ were probably due to the variable angle vibration of O-H. All these data indicate the saccharide property of AOP-w. Importantly, there was a distinct peak at 852 cm^−1^, which was likely caused by the deformation vibration of the C-H of α-conformation [27]. These results indicated that the α-linked residues were presented in the AOP-w.

### 3.3. Monosaccharide Composition of AOP-w

**Figure 2:**
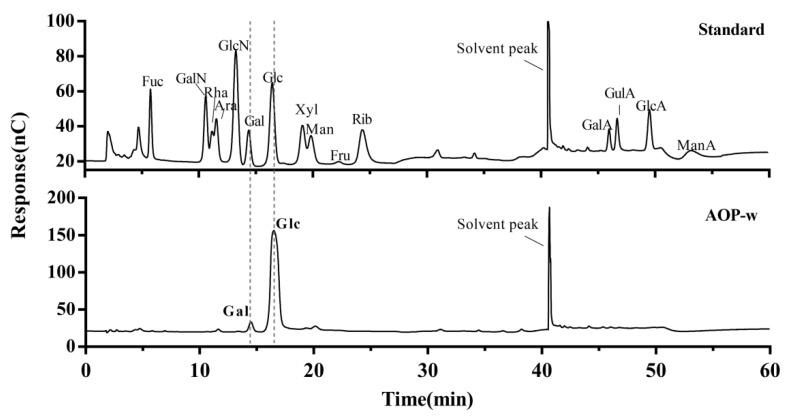
HPAEC chromatograms of mixed standards (**upper panel**) and AOP-w (**lower panel**) with gray dotted lines to indicate the same retention time.

After the hydrolyzation of polysaccharides at 120 °C for 3 h, the monosaccharides in AOP-w were obtained and determined by the HPAEC system. As shown in Figure 2, the mixed monosaccharide standards could be distinctly separated from each other. The chromatogram of AOP-w displayed predominantly two intense peaks at 14.48 min and 16.48 min, which could be identified to be galactose (Gal) and glucose (Glc), respectively, when matched to the chromatogram of the mixed monosaccharide standards. The molar ratio of Gal and Glc was roughly 0.12:0.88. Moreover, previous studies revealed that the absolute configuration of all the glucopyranosyl and galactopyranosyl was a D-configuration [28].

### 3.4. Glycosidic Linkage Analysis of AOP-w

**Figure 3:**
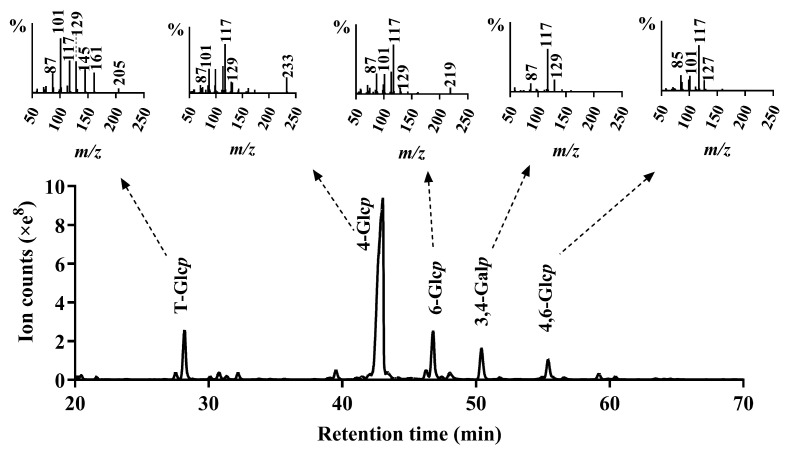
The GC chromatogram of PMAAs (**lower panel**) and the MS spectra of each PMAA (**upper panel**) of AOP-w after methylation.

To elucidate the glycosidic linkages of AOP-w, methylation analysis was performed, and the PMAAs were identified by GC-MS and are displayed in Figure 3 and Table 1. AOP-w is composed of glucose and galactose, as indicated by the monosaccharide composition analysis. Thus, the methylation results indicate that the glucose units of AOP-w are mainly bound at O-4, O-4,6 and O-6, demonstrated by the existence of the 2,3,6-Me_3_-Glc*p* (68.4%), 2,3-Me_2_-Glc*p* (4.5%) and 2,3,4-Me_3_-Glc*p* (10.4%) derivatives, respectively. In addition, the results also revealed a high quantity of non-reducing-end Glc*p* (10.3%) by the presence of 2,3,4,6-Me_4_-Glc*p*. Therefore, AOP-w consisted of backbone of (1→4)-linked Glc*p* units, substituted at O-6 by terminals of glucose. The galactose units of AOP-w were mainly bound at O-3,4, shown by the presence of 2,6-Me_2_-Gal*p* (6.4%). However, the branches located at the O-3 or O-4 position of galactose residues needed further NMR analysis.

It is well known that methylation analysis is not a quantitative method due to the lack of PMAA derivative standards. The molar ratios of each residue could only be roughly calculated. Our data showed that the molar ratio of the terminal glucose (10.3%) was roughly equal to the total ratios of the branching residues (10.9%) of (1→4,6)-linked Glc*p* (4.5%) and (1→3,4)-linked Gal*p* (6.4%).

### 3.5. NMR Analysis of AOP-w

NMR spectroscopy is recognized as a unique analytical technique for polysaccharide structure elucidation [29]. The NMR spectra of AOP-w were obtained and the chemical shifts of the glycosidic residues were annotated with an overall consideration of the methylation results, monosaccharide composition, NMR spectra and previous literature.

**Figure 4:**
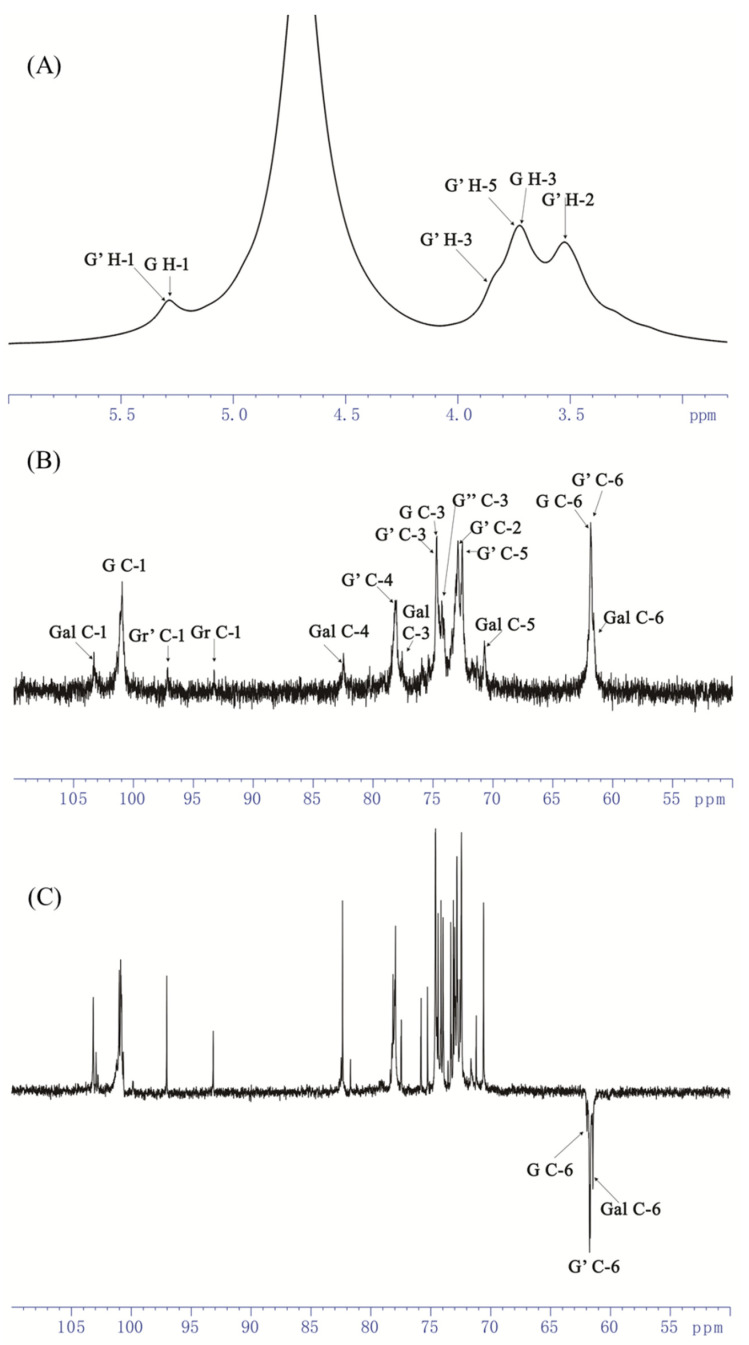
One-dimensional NMR spectra of AOP-w isolated and purified from the rhizomes of *A. officinarum*. (**A**) ^1^H NMR spectrum. (**B**) ^13^C NMR spectrum. (**C**) DEPT 135 ^13^C spectrum. Glc (G): T-α-Glc*p*; G′: 4-α-Glc*p*; G″: 4,6-α-Glc*p*; Gr and Gr′: reducing end α- and β-Glc*p*; Gal (Gal): 3,4-β-Gal*p*.

As shown in Figure 4, a distinct peak at δ5.31 was displayed within the anomeric region of the ^1^H NMR spectrum (Figure 4A), and several signals of the ring protons were presented in the region of δ3.2–4.4. The ^13^C NMR spectrum of AOP-w presented multiple signals at δ100–103 within the anomeric regions (Figure 4B), and the inverted peaks at the δ61–62 showed the -CH_2_ groups of C-6 of the residues (Figure 4C).

**Figure 5:**
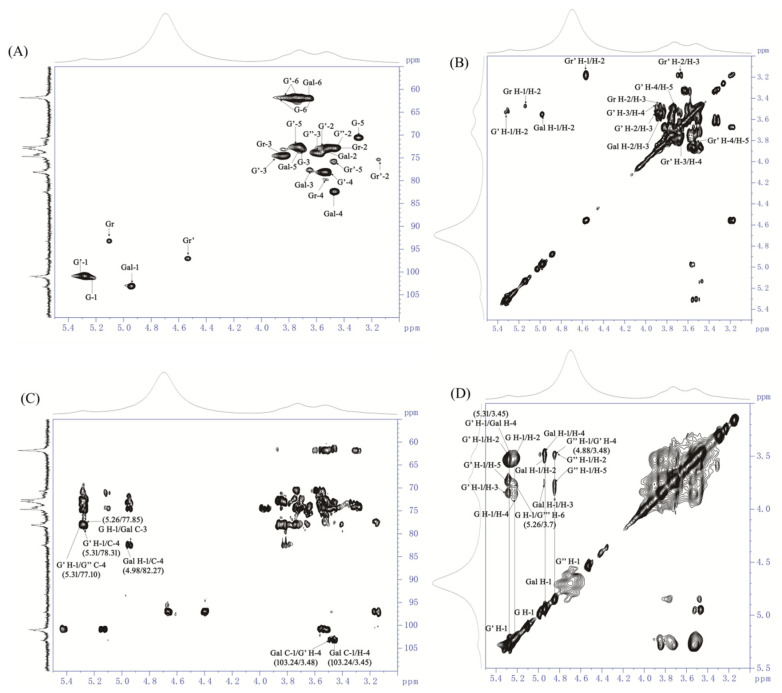
Two-dimensional NMR spectra of AOP-w isolated from the rhizomes of *A. officinarum*. (**A**) ^1^H/^13^C HSQC spectrum; (**B**) part of the COSY spectrum; (**C**) section of the HMBC spectrum with inter-residual proton/carbon atom correlations labeled; (**D**) the NOESY spectrum. Glc (G): T-α-Glc*p*; G′: 4-α-Glc*p*; G″: 4,6-α-Glc*p*; Gr: reducing end α-Glc*p*; Gr′: reducing end β-Glc*p*; Gal (Gal): 3,4-β-Gal*p*.

In addition, the cross peaks in the ^1^H/^13^C HSQC (heteronuclear single quantum coherence) correlation map were adopted to assign the H-C spin pairs and identified in Figure 5A. In total, the chemical shifts for five different residues were assigned and the other two residues were weakly detected in the anomeric region (Table 2), wherein δ5.26/101.33, δ5.31/100.89 was determined from the H-1/C-1 of α-D-Glc*p* units [30], and the two correlations at δ5.13/93.33 and δ4.58/97.01 were from the reducing end of Glc*p* units by comparison to the literature [29]. Moreover, the presence of a correlation at δ4.98/103.24, typical of H-1/C-1 of the β-D-Gal*p* unit [31], was observed in the anomeric region.

The ^1^H/^1^H COSY spectrum (correlation spectroscopy) gave information of the ring proton sequence. For the anomeric proton at δ 5.31, its ring proton signals were attributed according to the COSY spectrum. As shown in Figure 5B, the correlation peaks at δ 5.31/3.55, 3.55/3.9, 3.9/3.48 and 3.48/3.76 were clearly observed and readily assigned to H-1/H-2, H-2/H-3, H-3/H-4 and H-4/H-5. These chemical shifts of protons were further verified by the NOESY spectrum (Figure 5D). Furthermore, C-1 to C-5 could be assigned according to the coupling peaks in the HSQC spectrum. As a result, the anomeric δ_H/C_ 5.31/100.89 and its corresponding ring protons and carbons were assigned as δ_H/C_ 3.55/72.91, 3.90/74.56, 3.48/78.31 and 3.76/72.53, as listed in Table 2. Therefore, the C-4 (78.31 ppm) signal was shifted to the low field in a comparison with unsubstituted residues, indicating that the anomeric δ_H/C_ 5.31/100.89 was from the α-(1-4)-linked Glc*p* residue. Similarly, the anomeric δ_H/C_ 5.26/101.33 was from the terminal α-linked Glc*p* residue. The δ_H/C_ 4.88/99.89 and δ_H/C_ 4.92/99.10 were from the 1,4,6-α-Glc*p* residue and 1,6-α-Glc*p*, respectively, which were quite similar, implying the existence of linear 1,4-α-glucan branched at O-6.

In addition, the ^1^H NMR resonance assignments of the monosaccharide-reducing end of →4)-Glc*p* were readily performed by ^1^H/^1^H COSY. The ring H/C of the reducing end of →4)-α-D-Glc*p* was found in 3.41–3.86/61.6–79.5, clearly distinguishing it from the →4)-β-D-Glc*p* in 3.12–3.85/61.67–78.73. The observed chemical shifts corresponded closely with those previously reported [29].

The anomeric signal at δ4.98/103.24 was unambiguously attributed to β-D-Gal*p* unit, as determined by the HSQC and COSY spectra. Again, the ring protons of the β-D-Gal*p* unit were allocated from COSY, and the corresponding carbon atoms were assigned from HSQC as well. Thus, H-1/C-1 to H-5/C-5 were δ_H/C_ 4.98/103.24, 3.54/73.55, 3.77/77.85, 3.45/82.27 and 3.75/73.04. On the other hand, C-3 (77.82) and C-4 (82.27) were shifted to the downward field in comparison to the unsubstituted relevant residues, indicating that the β-D-Gal*p* was substituted at the O-3 and O-4 positions. Therefore, the anomeric δ_H/C_ 4.98/103.24 was attributed to the β-1,3,4-linked-Gal*p*.

Furthermore, the sequence of sugar residues of AOP-w was clearly determined by the inter-residue correlations in ^1^H/^13^C HMBC (heteronuclear multiple bound correlation) and supported by NOESY correlations. Strong inter-residual proton/carbon atoms at δ5.31/78.31 and δ5.31/77.10 were identified on the HMBC spectrum (Figure 5C), suggesting the existence of →4)-α-D-Glcp-(1→4,6)-α-D-Glcp-(1→, →4)-α-D-Glcp-(1→4)-α-D-Glcp-(1→ linkage residues. Similarly, couplings at δ3.48/103.24 were identified, which suggested that the residues →3,4)-β-D-Gal*p*-(1→ and →4)-α-D-Glc*p*-(1→ were assembled. Thus, the backbone of AOP-w was →3,4)-β-D-Gal*p*-(1→4)-α-D-Glc*p*-(1→4)-α-D-Glc*p*-(1→4,6)-α-D-Glcp-(1→. Moreover, the following couplings were observed on the HMBC spectrum: the coupling of H-1 at δ5.26 of the residue α-D-Glc*p*-1→ to the carbon atom from the C-3 at δ77.85 of residue →3,4)-β-D-Gal*p*-(1→. This way, the terminal Glc*p* residues were assembled to the O-3 of the →3,4)-β-D-Gal*p*-(1→.

The inter-residue correlations of the overlapping H-1 signals in residues →6)-α-D-Glc*p*-(1→, →4,6)-α-D-Glc*p*-(1→, and α-D-Glc*p*-1→ were inconclusive in the HMBC, but were resolved by the NOESY spectrum (annotated in Figure 5D). The NOE contact correlation at δ5.31/3.45 (G′-H-1/Gal H-4) confirmed that the →4)-α-D-Glcp-(1→ linked at O-4 of →3,4)-β-D-Gal*p*-(1→. A strong correlation at δ4.88/3.48 suggested the assemble of the →4,6)-α-D-Glc*p*-(1→ and →4)-α-D-Glc*p*-(1→ residues. The correlations at δ5.26/3.7 demonstrated that the terminal Glcp residues were assembled to the O-6 of the →6)-α-D-Glc*p*-(1→ unit.

**Figure 6:**
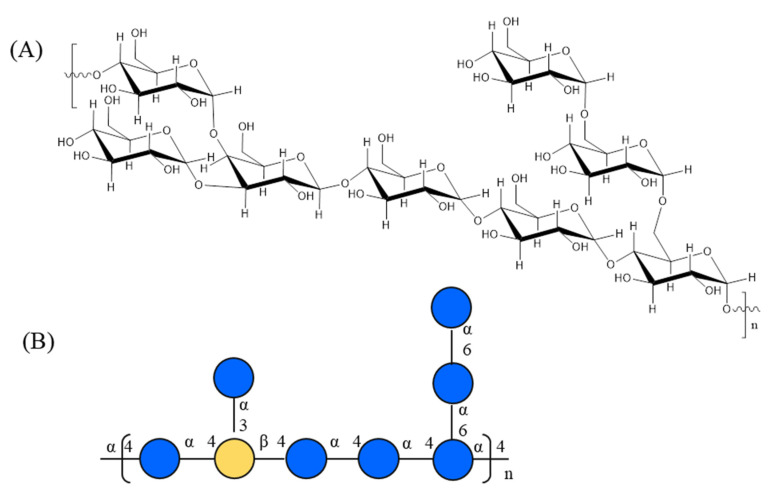
The putative chemical structure of AOP-w presented in (**A**) chair conformation and (**B**) colored dot diagram.

As a result, the chemical repeating unit of the *A. officinarum* polysaccharide was identified as galactoglucan. The backbone for the repeating units was composed of a α-1,4-linked-D-Glc*p* to β-1,4-linked-D-Gal*p*. The branches were attached to the main chain at the position of O-6 of the 1,4,6-linked Glc*p,* and O-3 of the 1,3,4-linked Gal*p,* as illustrated in Figure 6. However, the polysaccharides from *A. officinarum* have only been elucidated twice by Ni et al. [10] and Wen et al. [23]. In the former, they presented a glycan-type polysaccharide with linkage types of T-α-D-Glc*p*, (1,4)-α-D-Glc*p* and (1,4,6)-α-D-Glc*p*, which was a lack of fine-structure information. In the latter, they identified a pectic-type polysaccharide with homogalacturonan. Therefore, we identified, for the first time, galactoglucan in the structure of the AOP-w from *A. officinarum,* and described it in this study.

### 3.6. Immunoregulatory Activity of AOP-w

#### 3.6.1. AOP-w Increased the Cell Viability of RAW 264.7 Cells

**Figure 7:**
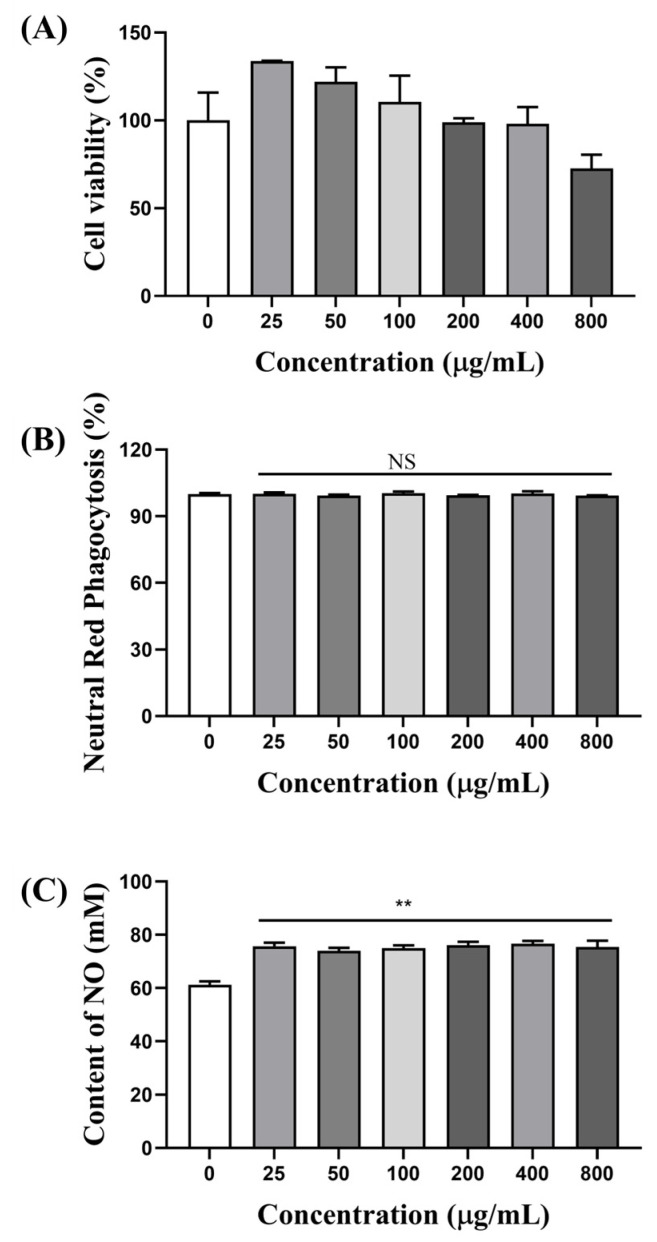
Immunoregulatory effects of AOP-w on RAW 264.7 cells. (**A**) Cell proliferation effects of AOP-w on RAW 264.7 cells determined by an MTT assay. (**B**) Phagocytosis effects of AOP-w on RAW 264.7 cells by neutral red uptake. (**C**) NO production of RAW 264.7 cells after AOP-w treatment for 24 h; ** *p* < 0.01 compared to the control group. NS represents no significance.

Macrophages are considered important target cells of polysaccharides [32]. They can not only recruit innate immune responses, but also be effector cells for anti-inflammation. RAW 264.7 cells are a generally recognized tool for macrophage mechanism investigation on regulating immunity. Therefore, RAW 264.7 cells were adopted to assay the immunoregulatory effects of AOP-w. The effects of AOP-w on the cell viability of RAW 264.7 macrophage cells were assayed using the MTT method, and the results are presented in Figure 7A. AOP-w was not cytotoxic to RAW 264.7 cells up to 400 μg/mL. When the concentration of AOP-w was within the range of 25–100 μg/mL, AOP-w could promote macrophage cell proliferation.

#### 3.6.2. Effects of AOP-w on Phagocytosis

The neutral red uptake index of AOP-w-treated RAW 264.7 cells was used to identify the phagocytosis properties of RAW 264.7 cells. As shown in Figure 7B, there was no significant difference of the phagocytosis index between the control and the AOP-w treatment groups, suggesting that the polysaccharides could not increase the phagocytic capacities of macrophages.

#### 3.6.3. AOP-w Promoted NO Secretion

Macrophages remove pathogens directly by phagocytosis and indirectly through pro-inflammatory factor secretion. The effects on the production of NO by AOP-w were determined. As shown in Figure 7C, the NO was significantly increased (*p* < 0.01) after AOP-w treatment for 24 h.

#### 3.6.4. Transcriptome Analysis Using RNA-Seq

**Figure 8:**
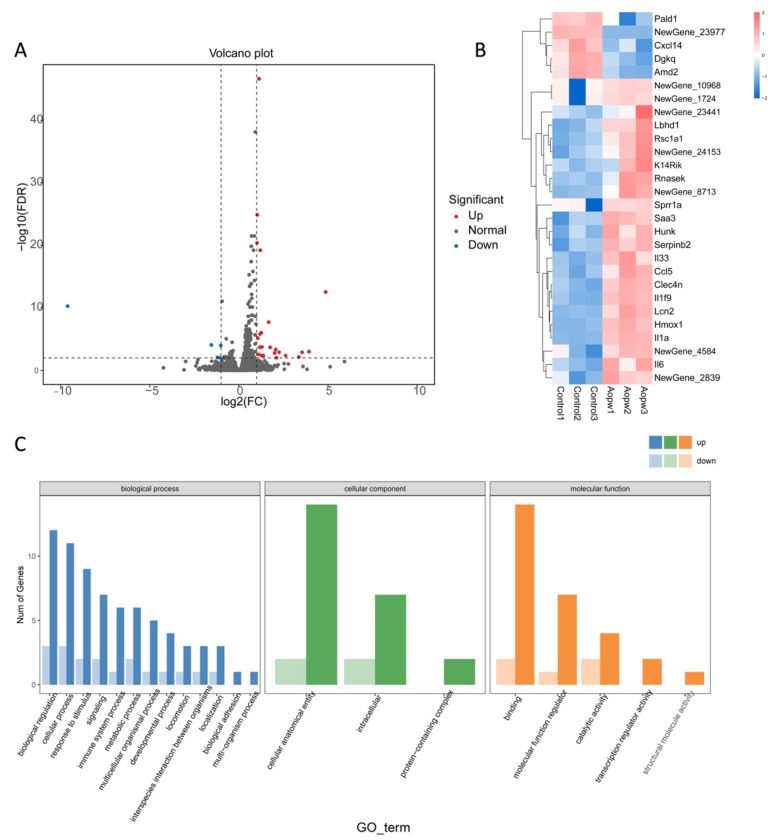
DEGs between the control and AOP-w-treated groups, and GO enrichment analysis. (**A**) Volcano plot. (**B**) Heatmap. (**C**) The GO enrichment analysis.

To further understand the immunomodulatory mechanism of AOP-w relating to RAW 264.7 cells, we conducted transcriptome analysis using RNA sequencing. Our analysis identified a total of 20 DEGs, of which 4 were downregulated and 16 were upregulated genes compared to the control group. These upregulated and downregulated genes are depicted in the volcano plot (Figure 8A), where blue represents the downregulated genes and red represents the upregulated genes. In Figure 8B, the DEG samples from the same treatment are clustered into categories among each other due to the similarity of their gene expression patterns. To investigate the biological functions of DEGs in the AOP-w treatment group compared with the control group, we utilized the GO database to obtain relevant functional annotations. Figure 8C shows the most significantly enriched GO terms for the molecular function, cellular component and biological process. Among the DEGs, the biological processes with the most enriched gene numbers are cellular anatomical entity, intracellular and protein-containing complex. As reported previously, the enriched GO terms in the biological processes of polysaccharides are commonly associated with the cellular anatomical entity and protein-containing complex [33].

#### 3.6.5. KEGG Pathway Enrichment Analysis

**Figure 9:**
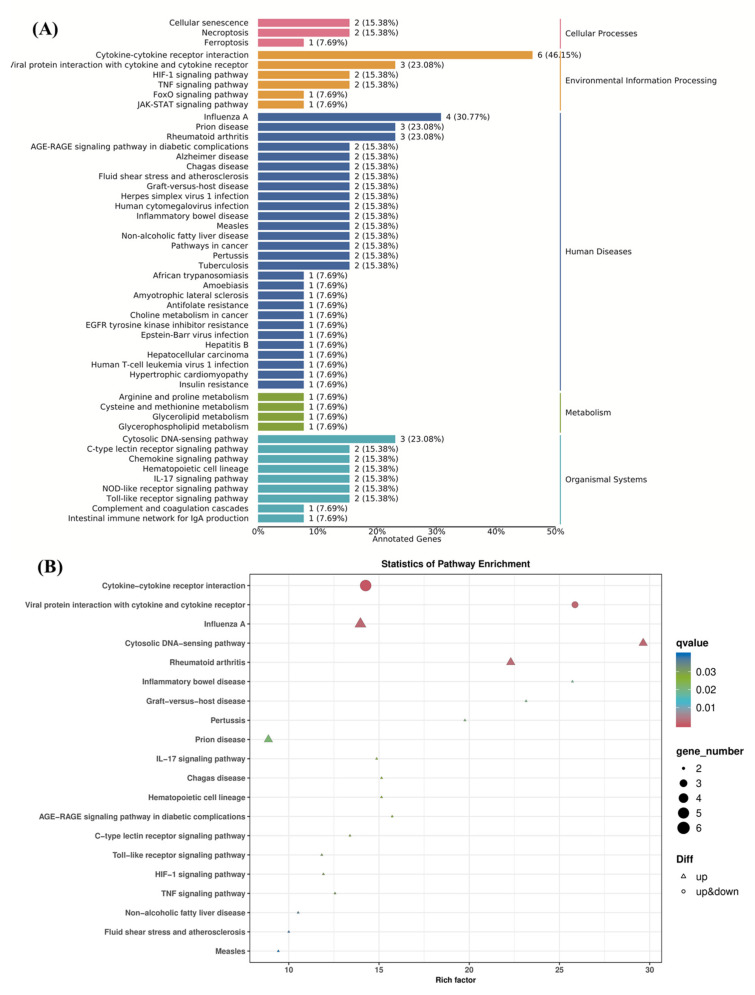
KEGG pathway enrichment analysis of DEGs comparing the control group and the AOP-w group. (**A**) Histogram of the KEGG pathway annotation and classification. (**B**) KEGG pathway analysis.

In order to screen out the potential signaling pathways related to AOP-w-induced macrophage activation, we conducted KEGG enrichment analysis. As illustrated in Figure 9A, the KEGG pathway database divides pathways into five groups, involving environmental information processing, metabolism, organismal systems, cellular processes and human diseases. The larger the enrichment factor, the higher the degree of enrichment, and the smaller the *p*-value, the more significant the enrichment. The candidate gene enrichment bubble chart (Figure 9B) shows 20 pathways with high DEG enrichment, among which are cytokine–cytokine receptor interaction, Toll-like receptor signaling and TNF signaling pathways. In addition, the significantly enriched DEGs are primarily distributed in categories such as environmental information processing, metabolism, human diseases, cellular processes and organismal systems, among which human disease is the most prominent category. These findings indicate that AOP-w may activate the TNF and NF-κB signaling pathways through interactions with Toll-like receptors, thereby affecting the immune modulatory activity of RAW264.7 cells.

## 4. Conclusions

In this study, polysaccharides from *A. officinarum*, a homology of medicine and food plant, were extracted, purified and structurally elucidated, and their biological activities evaluated. The Mw was calculated to be 5.26 kD. The homogeneity of the AOP-w was verified and purified AOP-w was obtained. The glucose and galactose units were AOP-w components identified at a molar ratio of 0.88:0.12. The methylation analysis of AOP-w highlighted the presence of terminal Glc*p*, 4-linked Glc*p*, 4,6-linked Glc*p*, 6-linked Glc*p* and 3,4-linked Gal*p*. Then, NMR data showed that AOP-w was a galactoglucan with →4)-α-D-Glcp-(1→3,4)-β-D-Gal*p*-(1→ in the backbone. AOP-W may activate the TNF and NF-κB signaling pathways by binding to Toll-like receptors, thereby affecting the immune modulatory activity of RAW 264.7 cells. With that, this study lays a solid foundation for further structure–function analyses of polysaccharides from *A. officinarum*.

## Figures and Tables

**Table 1:** The Methylation results of AOP-w.

RT	Methylated Sugar	Mass Fragments (*m*/*z*)	Molar Ratio	Linkage Type
28.203	2,3,4,6-Me_4_-Glc*p*	43, 71, 87, 101, 117, 129, 145, 161, 205	0.103	Glc*p*-(1→
43.028	2,3,6-Me_3_-Glc*p*	43, 87, 99, 101, 113, 117, 129, 131, 161, 173, 233	0.684	→4)-Glc*p*-(1→
46.793	2,3,4-Me_3_-Glc*p*	43, 87, 99, 101, 117, 129, 161, 173, 233	0.104	→6)-Glc*p*-(1→
50.395	2,6-Me_2_-Gal*p*	43, 87, 97, 117, 129, 149	0.064	→3,4)-Gal*p*-(1→
55.388	2,3-Me_2_-Glc*p*	43, 71, 85, 87, 99, 101, 117, 127, 159, 161, 201, 261	0.045	→4,6)-Glc*p*-(1→

**Table 2:** ^1^H and ^13^C chemical shifts of the polysaccharide-fraction AOP-w obtained from the NMR experiment. The substituted carbon atoms are in bold.

Glycosyl Residue	H1/C1	H2/C2	H3/C3	H4/C4	H5/C5	H6a,b/C6
α-D-Glc*p*-1→	5.26	3.6	3.7	3.95	3.17	3.6; 3.82
G	101.33	71.94	74.03	70.61	71.68	62.3
→4)-α-D-Glc*p*-(1→	5.31	3.55	3.9	3.48	3.76	3.71; 3.81
G′	100.89	72.91	74.56	**78.31**	72.53	61.89
→4,6)-α-D-Glc*p*-(1→	4.88	3.49	3.67	3.36	3.57	3.78; 3.89
G″	99.89	72.86	74.02	**77.10**	70.69	**68.46**
→6)-α-D-Glc*p*-(1→	4.92	3.53	3.67	3.47	3.86	3.70
G″’	99.10	72.73	74.63	70.87	71.47	**66.80**
→4)-α-D-Glc*p*	5.13	3.46	3.86	3.55	3.74	3.7; 3.79
Gr	93.33	72.8	74.67	**79.52**	72.11	61.67
→4)-β-D-Glc*p*	4.58	3.19	3.7	3.85	3.5	3.7; 3.75
Gr′	97.01	75.56	77.5	**78.39**	75.13	61.67
→3,4)-β-D-Gal*p*-(1→	4.98	3.54	3.77	3.45	3.75	3.75; 3.85
Gal	103.24	73.55	**77.85**	**82.27**	73.04	61.79

Note: ^1^H NMR chemical shift values are recorded with respect to the HOD signal at δ4.70 ppm at 25 °C, while ^13^C NMR chemical shift values are recorded with reference to d6-acetone at δ30.89 ppm at 25 °C.

## Data Availability

The original contributions presented in the study are included in the article, further inquiries can be directed to the corresponding author.
